# Laparoscopic right hemihepatectomy following a novel optimized portal vein embolization: a video case report

**DOI:** 10.1186/s12876-022-02321-x

**Published:** 2022-06-03

**Authors:** Lei Liu, Wenbin Ding, Xue Liu, Weiping Zhou, Shengxian Yuan

**Affiliations:** 1 The Third Department of Hepatic Surgery, Eastern Hepatobiliary Surgery Hospital, 225 Changhai Road, Shanghai, 200438 China; 2 The Department of Radioactive Intervention, Eastern Hepatobiliary Surgery Hospital, Shanghai, 200438 China

**Keywords:** Laparoscopic major hepatectomy, Hepatocellular carcinoma, Portal vein embolization

## Abstract

**Background:**

This article is the first report of laparoscopic major hepatectomy of Hepatocellular carcinoma (HCC) following optimized portal vein embolization (oPVE).

**Case presentation:**

The patient was diagnosed with a single 3 × 3.5 cm HCC located in segment 5 and 8 detected by enhanced computed tomography and magnetic resonance imaging. The lesion was adjacent to the right anterior and posterior portal veins, making it difficult to confirm the adequate liver functional remnant volume, surgical margin and R0 resection. In addition, the liver cirrhosis induced by a long history of chronic hepatitis B virus increased the potential risk of postoperative liver failure and refractory ascites. Therefore, we conducted a laparoscopic surgery following oPVE, by which the safe tumor margin was ensured and the outcome of the surgery was improved. The patient was discharged on the seventh day after the surgery. The AFP gradually decreased to a normal level during the 90-day follow-up.

**Conclusion:**

This case report demonstrates that, in experienced hands for selected patients, laparoscopic hepatectomy after portal vein embolization is feasible and may be an alternative to open liver resection.

**Supplementary Information:**

The online version contains supplementary material available at 10.1186/s12876-022-02321-x.

## Background

Laparoscopic hepatectomy as a therapy for treating liver tumors has become more common in recent years. Along with the development of imaging techniques, surgical devices and skills, laparoscopic liver resection has expanded its indication from minor to major hepatectomies such as right hemihepatectomy [1]. A series of studies have demonstrated a similar recurrence-free survival (RFS) and overall survival (OS) of HCC patients who underwent either laparoscopic liver resection (LLR) or open surgery [1–3]. Moreover, the advantages of LLR such as less compression damage to liver by its minimally invasive nature and faster recovery time bring more clinical benefits to patients [4].

The preoperative liver function and volume assessment was crucial to decrease the risk of morbidity and mortality of major hepatectomy [5]. PVE was thought to provide larger functional liver remnant volume when postoperative reserved liver volume shortage was expected [6] Moreover, it may help to guarantee the surgery margin > 1 cm, which is considered important for a favorable surgery outcome. Previous studies reported a median interval between PVE and surgery of 21–24 days [3, 6]. The potential drop-out rate of patients owing to insufficient liver hypertrophy and disease progression is the drawback of the conventional PVE. Alternatively, we applied an oPVE with complete occlusion of branch and main portal vein to acquire a more rapid regeneration of reserved liver. Given the above, we scheduled a laparoscopic right hemihepatectomy (An additional movie file displays the procedure in a more detailed way [see Additional file 1]) following oPVE. To our knowledge, there were no reports presenting outcomes of the laparoscopic major liver resection following optimized PVE.

## Case presentation

**Fig. 1:**
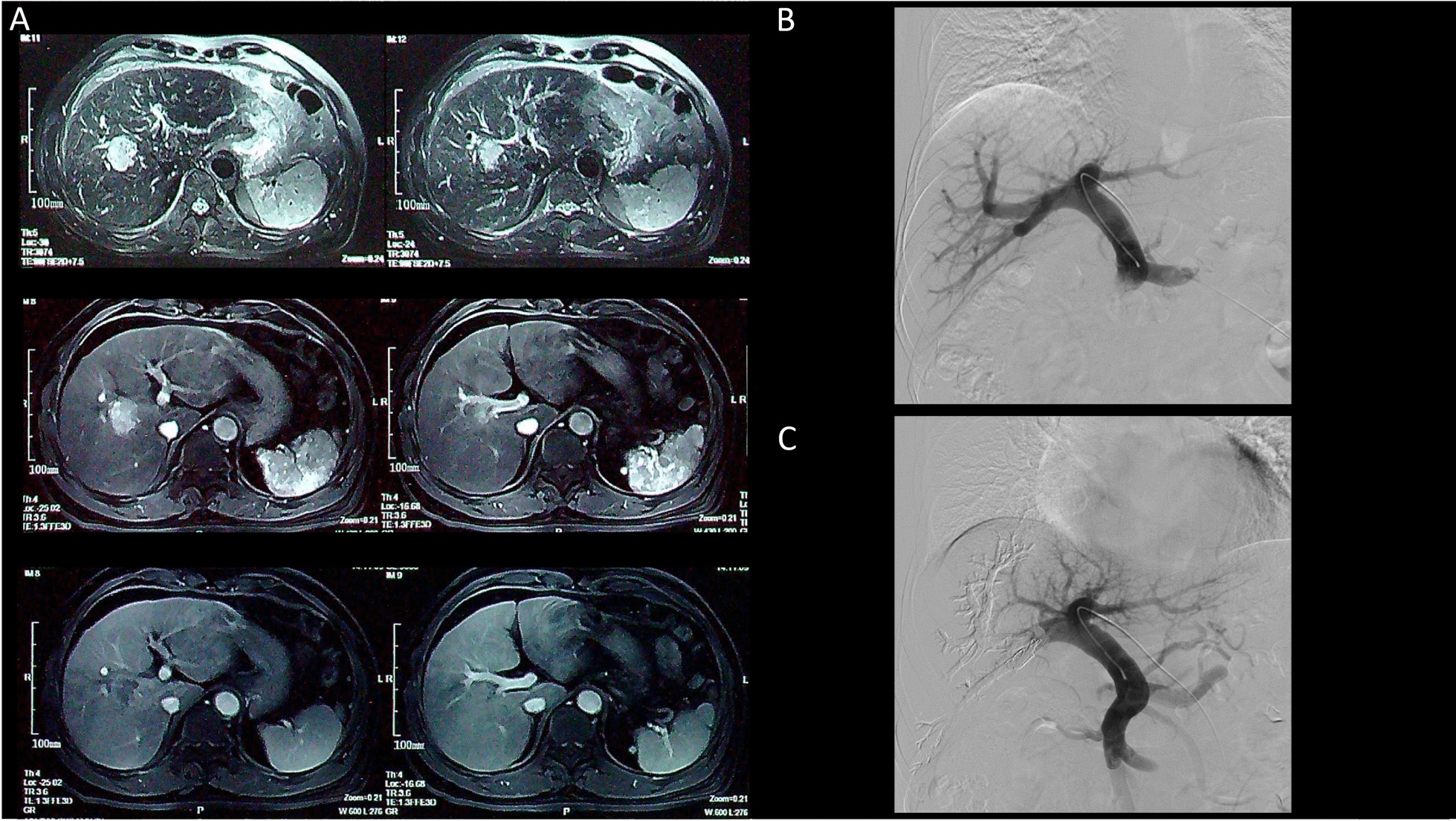
**A** Magnetic resonance imaging (MRI) before PVE in different phases; **B** Angiography of portal vein before embolism; **C** Angiography of portal vein after embolism

A 57-year-old male (height 173 cm, weight 75 kg) was admitted to our center with a diagnosis asymptomatic HCC. The lesion was located in segment 5 and 8, which was close to the right anterior and posterior portal veins (Fig. 1A). There was no other medical history but a 20-year history of HBV under a normal level of HBV-DNA.

Blood test showed a total bilirubin of 17.9 umol/L, ALT of 47 U/L, AST of 43 U/L, AFP of 175.8 ng/ml and PIVKA of 79 mAU/ml. Platelet count was of 180 × 10^9^/L and hemoglobin of 160 g/L. The 3-dimentional reconstruction examination gave the left hemiliver volume of 468 ml. The remnant liver volume to total liver volume (RLV-TLV) was 42.01% and the RLV to body weight ratio (RLV-BWR) was 0.6% [7, 8]. Based on the above, we considered it necessary to carry out hemihepatectomy to ensure R0 resection and adequate surgical margin, and PVE before the operation was essential to provide more remnant liver volume for surgical safety reason.

Optimized percutaneous transhepatic portal vein embolization (oPVE) was performed by contralateral approach under local and sedation anesthesia. After giving antibiotics, a periportal left vein was punctured to provide the access to the target vessels. The embolic material was n-Butyl-2-Cyanoacrylate (Compont, Beijing Compont medical devices CO., LTD.), iodized oil and Interlock coil (14 mm *30 cm, Boston Scientific Corporation). A microcatheter was put into the deepest position in the branch of the right portal vein through a 5F catheter. The Compont and iodized oil ratio was 1:3 to 1:4. The mixture was injected through the microcatheter. The same procedure was performed on the other branches and the interlock coil was put in the right portal vein. The operation time was 30 min. The portograms showed a well-performed embolization in the right portal vein and its branches (Fig. 1B, C). The patient had no other complications but complain of upper abdominal pain, and was discharged on the third day after the operation.

Nine days later, the patient revisited for further evaluation for the following resection. The left hemiliver volume increased significantly to 726 ml (RLV-TLV 56.80%; RLV-BWR 0.97%). AFP rose to 380 ng/ml and PIVKA was in the level of 90 mAU/ml. The size of the tumor remained almost unchanged. Based on the assessment of liver function and volume, the patient went through the surgery under the combined intravenous-inhalation anesthesia (CIVIA). The patient was placed in the spine position. The first trocar was inserted through umbilicus for laparoscope. One 5-mm and one 12-mm trocar were placed under the right costal arch for the main operator. One 10-mm trocar was placed under the right costal arch and one 5-mm trocar in the upper abdomen for the assistant. Firstly, the mobilization of liver included the cut of the ligament and the division of the adrenal gland. The right anterior interval was then exposed in front of the vena cava, the hepatic short veins were cut and ligatured. The pringle maneuver via a prepared tourniquet was performed. After the common cholecystectomy, the hilar plate was opened and the root of the right portal vein was exposed. Ligation of the vessel was done with a thread and the demarcation line could be seen on the liver surface. Along with the line, the liver parenchyma was opened widely until the right Glissonean pedicle was exposed. The Glissonean pedicle was cut by a linear stapler through the 12-mm trocar. The division was going on to the hepatic vein side to reveal the middle hepatic vein (MHV). The root of the right and middle hepatic vein (RHV & MHV) could be identified in the deep position along with the MHV. Finally, we used the linear stapler to dissect RHV and MHV at each origin and removed the resected liver. The incision below the right costal arch was extended to 7 cm, and the specimen was collected in a specimen bag and moved outside the body. The operation time was 185 min with blood loss close to 200 ml. (see Additional file 1: https://figshare.com/s/a31b1478f8e8f8f00e35) The surgical margin was negative and was 1.2 cm to the tumor. The patient was discharged on the seventh day without complications. The liver function became normal on the fourth day after the procedure.

## Discussion and conclusions

The insufficient future liver remnant (FLR) is always the Achilles heel of the major liver resection. Recently, many treatment strategies such as PVE, portal vein ligation (PVL), the associating liver partition and portal vein ligation for staged hepatectomy (ALPPS) and two-staged hepatectomy (TSH) have been chosen to increase FLR [9–11]. It seems that PVE can achieve a result of more liver volume with minor injury and lower postoperative morbidity and mortality based on previous studies [12]. Nevertheless, the disadvantage of conventional PVE described above still limited its application in the clinic. In the present case, we applied the mixture of iodized oil and n-Butyl-2-Cyanoacrylate instead of traditional embolic material such as microspheres and gelatin sponge particles. It provided better embolization and shorter interval, and the coil located in the right portal vein prevented the mixture from floating into the opposite side. The exposure time of radiation was around 5 min and the hospitalized cost was also lower than that of the conventional treatment.

In the past, PVE was always applied as a passive selection when insufficient FLR presented. In the present case, we actively carried out a PVE to chase a preferred surgical outcome. PVE is not only for huge tumor that leads to insufficient FLR, but can also be applied to small centrally seated tumor, for which enough surgical margin needs to be guaranteed [13, 14].

The preoperative liver 3-dimentional visualization technique can provide a more accurate assessment of liver volume. It is commonly used before major hepatectomy in our center [15]. Along with the improvement of laparoscopic surgical technique and devices, LLR has low postoperative morbidity and mortality, similar RFS and OS to open liver resection and short hospitalization [1, 3, 4]. In LLR following the oPVE, there are several points that need to be given attention. First of all, the inflammation and morphological change of the liver caused by PVE might have negative impact on the surgery procedure. Secondly, it is very important to determine the position of coils in the portal vein via CT or MRI before LLR. In the present case, the coils were distal to the bifurcation of the portal vein, and the right portal pedicle could be cut by the linear stapler directly, however, if the coils were close to the bifurcation, an intrathecal procedure should be conducted: open the right portal vein, clean up the coils and suture the vessel ends.

Laparoscopic right hemihepatectomy following oPVE is safe and feasible for selected patients. Minimally invasive procedure, shorter interval between oPVE and LLR and shorter hospitalization together make it acceptable to patients. In the future, it has the potential to become an effective alternative to traditional open liver resection.

## Supplementary Information

**Additional file 1**: The video of laparoscopic right hepatectomy. A laparoscopic right hepatectomy following a novel portal vein embolism was performed.

## Data Availability

The datasets used and/or analyzed in the current study are available from the corresponding author upon reasonable request.

## Abbreviations

HCC: Hepatectomy of Hepatocellular carcinoma
oPVE: Optimized portal vein embolization
CT: Computed tomography
MRI: Magnetic resonance imaging
HBV: Hepatitis B virus
RFS: Recurrence-free survival
OS: Overall survival
LLR: Laparoscopic liver resection
RLV-TLV: Remnant liver volume to total liver volume
RLV-BWR: The remnant liver volume to body weight ratio
CIVIA: Combined intravenous-inhalation anesthesia
RHV & MHV: The right and middle hepatic vein
FLR: Future liver remnant
PVL: Portal vein ligation
ALPPS: The associating liver partition and portal vein ligation for staged hepatectomy
TSH: Two-staged hepatectomy
TACE: Transarterial chemoembolization
